# Electrical Effects of Sprays

**Published:** 1896-04

**Authors:** 


### Electrical Effects of Sprays.

A correspondent, says *Appleton's Popular Science Monthly*, writing to us concerning the effect of various atmospheric conditions on health and bodily vigor, cites his own experience in a fire-brigade as having led him to believe that deficiency of ozone and other unfavorable conditions, and the effect of atmospheric impurities may be alleviated by inhalation through a spray of cold water. A method of ventilation of railroad cars which was very comfortable to passengers riding in cars so treated, but has been disused, depended upon the application of this principle. Its value is further confirmed by what Prince Krapotkin has said in one of his recent articles on current science concerning the theory of the development of electricity by spattering water. A few years ago Herr Lenard undertook a series of observations in Switzerland, on the electrical effect of waterfalls. It appeared that even small cataracts, only a few feet high, send into the air considerable charges of electricity, provided they bring down a large amount of rapidly dashing water. The smallest jets of water that drip on the rock sides, and even roaring streamlets, have the same effect. He suggested that the chief cause of electrification, is the tearing asunder of the drops of water as they fall on the wet surfaces at the bottom of the waterfall. The experiments on which these views are founded accord with the demonstration by Lord Kelvin and Messrs. Maclean and Goto that air, even absolutely dust-free, can be electrified by a jet of water. This source of electrification is further shown to be by no means insignificant, and the amount of electricity sent into the air in this way is immense. The importance of these facts in the economy of nature, says Prince Krapotkin, is self-evident. "The supply of electricity in the air is continually renewed. The waterfalls in the valleys, the splashing of the waves on the shores of lakes and rivers, and the splash of drops of rain on the ground send masses of negative electricity into the air; even the watering of our streets, and of our plants in the orchards has the same effect on a limited scale. On the other side, the waves of the sea, as they break against the rocks and fall back in millions of droplets upon the beach, supply the air with masses of positive electricity the amount of which rapidly increases after each storm. And when we stand on the sea-beach, we not only inhale pure ozonified or iodized air; we are, so to say, surrounded by an electrified atmosphere, which, as already remarked by Humboldt and often confirmed since, must have a stimulating effect upon our nervous activity as well as upon the circulation of sap in plants."

